# Ensemble-Based Bounding Box Regression for Enhanced Knuckle Localization

**DOI:** 10.3390/s22041569

**Published:** 2022-02-17

**Authors:** Ritesh Vyas, Bryan M. Williams, Hossein Rahmani, Ricki Boswell-Challand, Zheheng Jiang, Plamen Angelov, Sue Black

**Affiliations:** School of Computing and Communications, Lancaster University, Lancaster LA1 4YW, UK; h.rahmani@lancaster.ac.uk (H.R.); r.boswell-challand@lancaster.ac.uk (R.B.-C.); z.jiang11@lancaster.ac.uk (Z.J.); p.angelov@lancaster.ac.uk (P.A.); sue.black@lancaster.ac.uk (S.B.)

**Keywords:** knuckle localization, object detector, ensemble, forensics

## Abstract

The knuckle creases present on the dorsal side of the human hand can play significant role in identifying the offenders of serious crime, especially when evidence images of more recognizable biometric traits, such as the face, are not available. These knuckle creases, if localized appropriately, can result in improved identification ability. This is attributed to ambient inclusion of the creases and minimal effect of background, which lead to quality and discerning feature extraction. This paper presents an ensemble approach, utilizing multiple object detector frameworks, to localize the knuckle regions in a functionally appropriate way. The approach leverages from the individual capabilities of the popular object detectors and provide a more comprehensive knuckle region localization. The investigations are completed with two large-scale public hand databases which consist of hand-dorsal images with varying backgrounds and finger positioning. In addition to that, effectiveness of the proposed approach is also tested with a novel proprietary unconstrained multi-ethnic hand dorsal dataset to evaluate its generalizability. Several novel performance metrics are tailored to evaluate the efficacy of the proposed knuckle localization approach. These metrics aim to measure the veracity of the detected knuckle regions in terms of their relation with the ground truth. The comparison of the proposed approach with individual object detectors and a state-of-the-art hand keypoint detector clearly establishes the outperforming nature of the proposed approach. The generalization of the proposed approach is also corroborated through the cross-dataset framework.

## 1. Introduction

**Figure 1:**
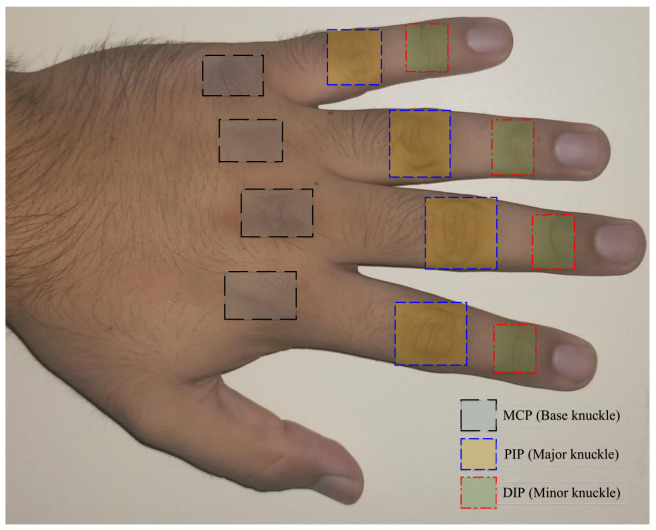
Depiction of MCP, PIP, and DIP joints on dorsal view of hand.

Forensic science has largely relied upon face and fingerprint modalities when it comes to identifying the perpetrators of serious crimes. However, it is not always feasible to obtain any sample of these traits with acceptable quality, or at all. In fact, there are instances when images of the hand were the only available evidence to compare against the identity of the perpetrator [1,2]. The texture and creases of the finger-knuckles, which are visible on the dorsal view of the hand, can prove vital in human identification [3]. The knuckles are formed on three types of finger joints which are termed as the metacarpophalangeal (MCP), proximal interphalangeal (PIP), and distal interphalangeal (DIP) joints, which in the biometrics terminology are also known as the base, major, and minor knuckles, respectively [4]. The knuckle nomenclature is depicted in Figure 1. It is also pertinent to specify here that the current work only considers the knuckles from the four fingers, and not from the thumb (which only has MCP and interphalangeal (IP) joints).

The knuckle patterns have been extensively used for biometrics applications in the last decade [3,5,6,7,8,9,10,11,12]. The initial attempts of knuckle recognition employed only the PIP joints (or major knuckles), as they tend to possess relatively larger area comprising knuckle creases and corresponding texture [3]. The state-of-the-art methods to extract the knuckle features included tools such as Gabor [8] and log-Gabor filters [13], Radon transforms [3,6], and phase-only correlation [14]. With the progression of technology, efficient methods had been derived to recognize the humans by virtue of their DIP (minor) [9] and MCP (base) [4] knuckles as well.

Notably, there are very limited works in the literature that elaborate on the automated localization of knuckle regions [8,15,16]. On one hand, the work by Zhang et al. [8] utilized a contact-based acquisition setup which comprised of a finger bracket and a CCD camera to capture the knuckle region of the index and middle fingers of human hands. The acquisition was followed by extraction of the region of interest (ROI) in an automated way, using the edge detection and convex direction coding scheme. The other works [15,16], on the other hand, employed automatic localization of key points from the hand-contour images by finding the finger-valleys. These valley keypoints were then used to localize various knuckle regions (MCP, PIP, and DIP). However, the keypoint localization approach suffers from an inherent limitation that arises due to its demand to have wide-opened fingers. Furthermore, forensic applications require expert anthropologists for adept localization of knuckle creases, leading to the issues related to long annotating time and the subjectivity involved. These factors also act as a bottleneck for evaluation of large datasets.

Simon et al. [17] proposed Openpose as a state-of-the-art real-time hand keypoint detector, which used multiview bootstrapping process to detect 21 keypoints from the input hand image. The Openpose detector utilized the modified architecture of convolutional pose machines (CPMs) which in turn was used to predict the confidence map for each keypoint of the hand. The Openpose keypoint detector worked with an input hand image of dimensions 368 × 368, assuming a bounding box around the right hand. The trained model for Openpose hand keypoint detector is made publicly available (URL: https://github.com/CMU-Perceptual-Computing-Lab/openpose/releases, accessed on 21 December 2021) and can readily be used to compare relevant works.

Deep learning (DL) based approaches of object detection are capable of detecting the objects irrespective of their shape, scale, background, and orientation [18]. The tremendous competence of these DL based approaches can be utilized for the task of knuckle localization with high accuracy, despite the challenges such as variations in sizes of the (MCP/PIP/DIP) knuckles, the backgrounds, the orientations (arbitrary angles due to rotation of hands), and the poses (opened/closed fingers). Moreover, the availability of high computing power can facilitate the deployment of DL based knuckle region detectors for larger datasets. However, evidence suggests that such DL based approaches suffer from data scarcity (which is quite common in the biometrics applications) and inabilities of the single detector model, but usage of data augmentation schemes and ensemble of multiple object detectors could solve these issues to a large extent. Among these two solutions, the data augmentation can be an ingenious step, whereas performing ensemble of various detectors can be a complex and sophisticated task. In this paper, we present an ensemble approach for automated localization of the knuckle regions, which leverage from the capabilities of individual object detectors. In order to make the investigations more comprehensive, we choose the individual detectors from both the single-stage and two-stage detector classes (YOLO and Faster R-CNN, respectively [19]).

The major contributions of our work can be listed as follows:

1. The ground truth bounding boxes (GTBBs) for the MCP, PIP, and DIP knuckles from a subset of one thousand images each from two public-domain datasets are created and are being released (http://wp.lancs.ac.uk/icvl/research/datasets/, accessed on 21 December 2021) with this paper. We believe that this ground truth will serve as an aid to the researchers willing to reproduce/advance our results.
2. An improved way of automated knuckle localization is presented, which utilizes the region predictions from multiple object detector models and combine those predictions through an ensemble approach. This ensemble approach is tested on two large-scale public hand dorsal databases (HD and 11k) and one unconstrained hand dorsal database (H-Unique).
3. A capacious evaluation is employed to demonstrate the potential of the proposed approach for both localization and identification.
  - The evaluation of localization is performed through box-form and point-form analyses using metrics such as “suitable for identification”, “mean-IOUs”, and “inscribing accuracy values” (for full, half, and quarter ground truth boxes, respectively).
  - The proposed approach is compared against state-of-the-art Openpose hand keypoint detector, where it has demonstrated its outperforming nature for the problem at hand.
  - The potential of improvements in matching (or identification) evaluation is also demonstrated through a standard metric equal error rate (EER).
4. The generalization of the proposed approach is demonstrated through several cross-dataset or external evaluation experiments.

## 2. Proposed Methodology

This paper employs convolutional neural network (CNN) based object detection models for the task of knuckle localization. As stated in the introduction section, traditional ways of knuckle localization require certain hand-poses and illumination/background, whereas the hand images obtained as real evidence are not supposed to possess uniform backgrounds and/or specific hand poses. Hence, the deep learning based object detectors can serve the purpose of knuckle localization in a more robust and generalized way.

There are multiple object detectors, which could be employed for the said task. The popular CNN-based object detectors can be categorized as single-stage and two-stage detectors. On one hand, single-stage detectors such as You Only Look Once (YOLO) [20] can be deployed as a true end-to-end approach. On the other hand, there are two-stage detectors such as region-based convolutional neural network (R-CNN) [21], which generates the region of interest (through the region proposal networks) in the first stage followed by classification and bounding box regression in the second.

In this section, we first summarize the individual object detectors used in this work and then will demonstrate the employed ensemble approach. The proposed framework considers four individual detectors named as YOLO-v2, YOLO-v3, and Faster R-CNN with background models as ResNet50 and ResNet101. Among these four detectors, first two belong to the single-stage object detectors, whereas the last two models belong to the two-stage detectors category. Both category models are chosen to add generalizability to the proposed knuckle localization framework, facilitating an appropriate blend of speed (from the single-stage detectors) and accuracy (from the two-stage detectors), hence making the proposed ensemble framework more effective.

### 2.1. YOLO Detectors

You Only Look Once (YOLO) is a single-stage object detector where the bounding box predictions are made directly from the input image [20]. YOLO was originally proposed for real-time object detection with the capability to generate 100 bounding boxes per image. The YOLO pipeline works as a regression problem, where the class probabilities and bounding boxes are predicted from the small patches generated from the input image [18]. For the current work, we have chosen two improved versions of YOLO, which are YOLO-v2 and YOLO-v3, known for their certain advancements over the baseline YOLO methods.

The YOLO-v2 model improves the network’s convergence by adding the batch-normalization layer ahead of each convolutional layer. This add-on would also regularize the base model. Another improvement in the YOLO-v2 model was achieved by removing the fully-connected layer from the basic YOLO network and incorporating the mechanism of using anchor-boxes as reference to generate the box-predictions [22]. The other notable improvements which YOLO-v2 incorporated included dimension clusters, fine-grained features, and multi-scale training [18]. On the other hand, YOLO-v3 further improves over YOLO-v2 through the inclusion of objectness score of each prediction (obtained through logistic regression) in addition to the class probability and the bounding-box coordinates [23]. In addition to that, the bounding boxes are predicted in YOLO-v3 at three different scales through a deeper and robust feature extractor.

### 2.2. Faster R-CNN Detectors

The region-based convolutional neural networks (R-CNN) are the two-stage object detectors that exhibit high performance in the object detection tasks [21]. In addition to the baseline R-CNN, there exist two other variants, which are named as Fast R-CNN [24] and Faster R-CNN [25]. The basic modules of an R-CNN model are the category-independent region proposals, convolutional-network-based feature extractor, class-specific support vector machines (SVMs), and bounding box regressor [18]. For higher detail, R-CNN uses the selective search algorithm [26] for generating the region proposals, followed by extraction of fixed-dimension features and SVM classification.

In R-CNN, a forward pass of ConvNet is performed for each region proposal without sharing the computation, hence making the detection slow. To avoid this, the Fast R-CNN model was proposed, which used an RoI pooling layer to extract a fixed-length feature vector from the feature map of the whole image followed by inputting the feature vector to two sibling output branches softmax and bounding box regressor [24]. Apart from these improvements of R-CNN, Fast R-CNN still used selective search to generate the region proposals, which limited the overall training and testing speed. Hence, another improved version, called Faster R-CNN, was proposed, which replaced the selective search by a novel region proposal network (RPN) which could predict the regions with varying scales and aspect ratios. This RPN used to share the features with the detection network. Owing to its overall advantages, we employ Faster R-CNN for our knuckle-localization experiments. Another important aspect for the Faster R-CNN model is the backbone network, which acts as the feature extractor and generates the feature maps for the input images. The backbone networks are generally the classification networks with their last fully connected layer removed [18]. Moreover, deeper and densely connected backbone networks can be employed to achieve more competitive object detecting accuracy with Faster R-CNN [25]. The better features, which may be extracted from deeper backbone networks, could help in improved region proposals and eventually in higher detecting accuracy. It is for this reason we have considered two deeper backbone networks, named ResNet50 and ResNet101 [27], to explore their accuracy for the specific task of knuckle localization.

### 2.3. Weighted Box Fusion

Both the single-stage and two-stage object detectors work considerably for the knuckle localization problem. However, they both possess certain limitations in terms of detecting accuracy and the generalizability. Hence, in this paper, we present an ensemble approach, which can leverage from the individual advantages of the single- and two-stage detectors and generate the knuckle localizations in a more generalized and accurate way. For this purpose, we incorporate the weighted box fusion (WBF) scheme, which is meant for fusing the bounding-box predictions from individual detectors and producing the fused boxes and their final confidence scores [28]. The WBF approach is claimed to perform better than other popular approaches such as non-maximal suppression (NMS).

The WBF scheme works to combine the bounding box coordinates of various single object detectors (such as YOLO and Faster R-CNN), weighted with their corresponding confidence values, and produce the coordinates for the fused bounding box, which tends to be more indicative and inclusive when compared against the original predictions [28]. The scheme initiates with tracing down all the available prediction boxes from multiple detectors and forms the clusters of matching boxes (i.e., boxes which have higher intersection over union (IoU) values). The boxes from all such clusters are then fused together using the following equations [28]:

(1) $$\begin{array}{c} c_{f}=\frac{\sum_{b=1}^{B}c_{b}}{B}\\ x_{1,f}=\frac{\sum_{b=1}^{B}c_{b}\times x_{1,b}}{\sum_{b=1}^{B}c_{b}},x_{2,f}=\frac{\sum_{b=1}^{B}c_{b}\times x_{2,b}}{\sum_{b=1}^{B}c_{b}}\\ y_{1,f}=\frac{\sum_{b=1}^{B}c_{b}\times y_{1,b}}{\sum_{b=1}^{B}c_{b}},y_{2,f}=\frac{\sum_{b=1}^{B}c_{b}\times y_{2,b}}{\sum_{b=1}^{B}c_{b}} \end{array}$$

where *B* denotes the number of matching boxes (i.e., the number of boxes available for fusion) and $c_{b}$ is the confidence value for $b^{th}$ box. The bounding box coordinates are represented by their top-left ($x_{1,b},y_{1,b}$) and bottom-right ($x_{2,b},y_{2,b}$) corner points which, when fused through the WBF Equation (1), yield the corresponding coordinates ($x_{1,f},y_{1,f}$ and $x_{2,f},y_{2,f}$) for the fused box. After obtaining the fused confidence value ($c_{f}$), it is rescaled by the ratio of the number of boxes in a cluster to the number of models. This is essential, because sometimes the number of boxes in the cluster is small, indicating that only a few models have predicted it and hence there is a need to decrease the confidence [28].

## 3. Experimental Setup and Performance Metrics

The proposed framework of knuckle localization is tested with two large and public hand databases, known as PolyU hand-dorsal (HD) dataset [16] and the 11k dataset [29]. Both these datasets provide hand dorsal images with different backgrounds and poses. In addition to the public datasets, this paper presents evaluation with a third hand dataset, known as the H-Unique dataset (HUQ), which provides hand dorsal images with random backgrounds, illuminations, and orientations. More details about the datasets are furnished in the subsequent subsections.

### 3.1. HD Dataset

The HD dataset provides 4650 hand-dorsal images from right hands of 501 different subjects. All images in this dataset have a resolution of 1600×1200 and possess the same hand pose with all fingers wide open.

### 3.2. 11k Dataset

The 11k dataset provides 11,076 hand images in total, out of which 5680 images have dorsal view of hands. Further, these hand dorsal images belong to both left (2788 images) and right (2892 images) hands of 190 subjects. The resolution of images from 11k is identical to that of images from HD dataset. However, images from 11k possess varying hand poses such as opened/closed fingers and arbitrary orientations. Additionally, the hand dorsal images from both these datasets offer certain challenges in the form of accessories such as rings.

### 3.3. HUQ Dataset

**Figure 2:**
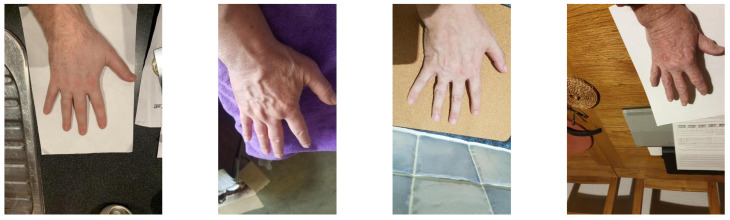
Sample images from the HUQ dataset.

This is an ambitious dataset being collected under the aegis of the H-Unique project (More details at https://h-unique.lancaster.ac.uk/, accessed on 21 December 2021). The dataset has more than 3000 hand dorsal images from people of various ethnic backgrounds. This dataset has the most challenging settings among all the three employed datasets in this paper as it is an unconstrained dataset where there is no control over the attributes such as backgrounds, orientations, scaling and illumination. A few sample images from the HUQ dataset are illustrated in Figure 2, which shows the lack of constraints and huge variations in the capturing conditions. Hence, we believe this dataset to be the best one to closely mimic the real-life scenarios of an actual crime scene.

In order to train the object detector models efficiently and perform the first-of-its-kind numerical evaluation of knuckle localization, we create a ground truth of a small subset of images from both the employed datasets. This ground truth comprises of 1000 images from each of the three employed databases. In order to make the ground truth representative of the entire dataset, random images from each class of the 11k and HD datasets are carefully included in it. Subsequently, manual annotations from experienced human annotators are acquired to complete the ground truth, wherein twelve annotations are made for each hand; labeling three knuckle regions (MCP, PIP, and DIP) for each of the four fingers. We look forward to releasing the ground truth data in the public domain through an accessible URL (will be provided on acceptance of the article), so that interested researchers could compare their results and/or advance the topic. Notably, the first 500 images from the ground truth are used to train the detector models, whereas the remaining ground truth images are utilized for testing and evaluation.

The proposed knuckle localization scheme is evaluated through several meaningful and readily applicable performance metrics. The evaluation is mainly performed with two types of analysis, namely box-form analysis and point-form analysis. Both these analyses are meant to measure the appropriateness of the detected knuckle regions with respect to the ground truth. The box-form analysis is targeted towards measuring the intersection over union (IoU) value of each predicted knuckle region with their corresponding ground truth box. Subsequently, these IoU values are then utilized to infer whether the predicted knuckle boxes are “acceptable for identification” or not. Another important measure in the box-form analysis is the “mean-IoU” which indicates the overall box-fitting ability for any object detector. On the other hand, the point-form analysis is meant for measuring the placements in terms of the centroids of the bounding boxes. In other words, we use gradual measures to indicate the preciseness of the centroids with respect to the full, half, and quarter ground truth bounding boxes, where quarter box measurement is apparently the most strict measurement and is expected to be the lowest in value. Another important advantage of the point-form analysis is that it enables us to compare our approach with an off-the-shelf hand keypoint detector called Openpose. More details about the definition and effectiveness of these metrics are provided in the following sections.

## 4. Results and Discussion

**Figure 3:**
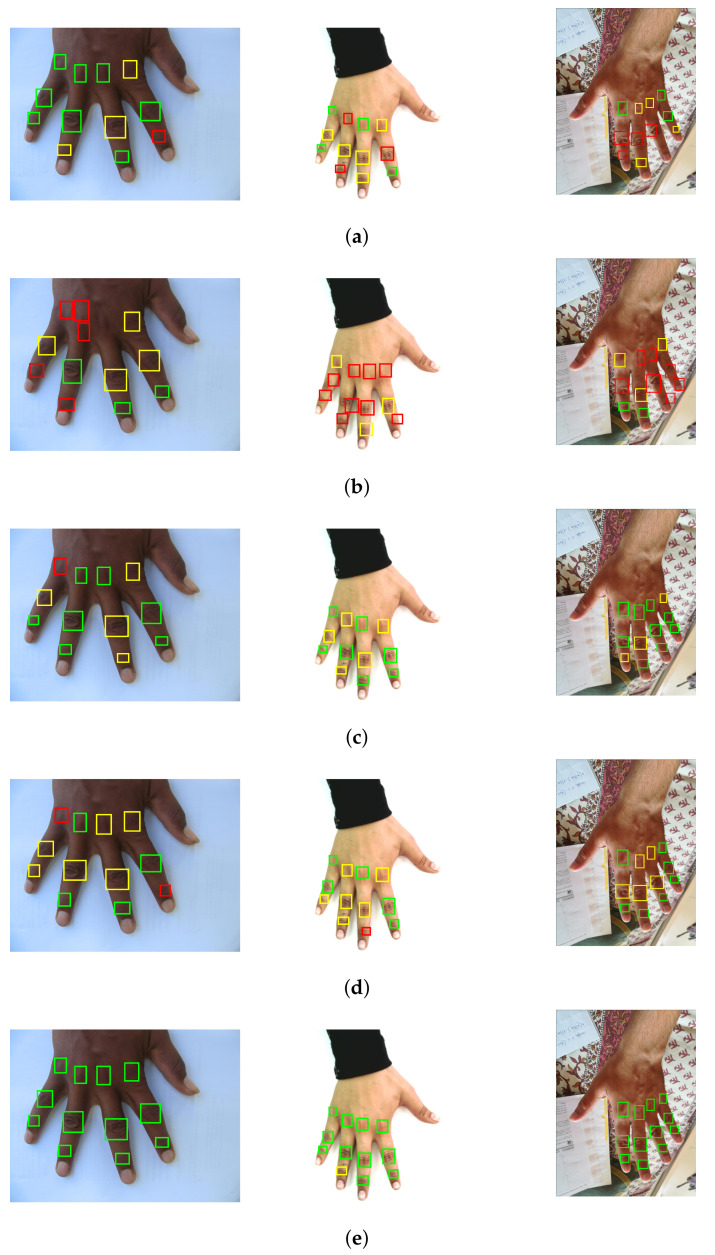
Samples showing knuckle localization for various approaches; for (left-column) HD, (middle-column) 11k, (right-column) HUQ dataset. Different colored boxes correspond to different types of regions: Green: Acceptable; Yellow: Borderline; and Red: Unacceptable knuckle predictions (refer to the web version for colors). Predictions are shown for (**a**) Yolo-v3, (**b**) Yolo-v2, (**c**) Faster R-CNN (ResNet50), (**d**) Faster R-CNN (ResNet101), and (**e**) WBF approach.

This section elucidates the various analysis performed to evaluate the results of the proposed knuckle localization. To the best of our knowledge, this is the first attempt to present the knuckle localization results in numerical form. These numerical results are obtained from the test subset of the ground truth formed from the employed hand datasets. In addition to the numerical results, Figure 3 demonstrates the localization results in pictorial form. This figure shows sample images from HD, 11k, and HUQ datasets, along with the bounding box predictions made from various individual object detectors and the proposed ensemble approach. For each prediction image, the predicted boxes are presented through bounding boxes with different colored boundaries. The colors are used to better illustrate the knuckle region predictions in the three categories, i.e., acceptable, borderline, and unacceptable, on the basis of their corresponding proportion of overlap with the ground truth boxes. It can be noticed from the figure that the box predictions made from the ensemble approach are more appropriate and cover the corresponding knuckle creases to a larger extent (which is also reflected from the IoU results shown later in this section).

### 4.1. Box-Form Analysis

As stated earlier, the box-form analysis of the knuckle localization results deals with the corresponding IoU values of the predicted boxes. It also defines whether the predicted knuckle can be used for biometric/forensic identification (a first-of-its kind dedicated evaluation metric). Hence, for finding the “suitable for identification” knuckle regions, there is a need of an appropriate threshold which should be chosen in an empirical way. For the current work, this threshold is chosen through visual observation of random images from the test set of both the employed datasets. Firstly, the identifiable knuckles were observed visually and then the correlation was obtained between the acceptability of the regions and their corresponding IoUs. Following this visual inspection, the IoU thresholds for both the hand datasets are fixed as 0.5 for the PIP (or major) knuckles and 0.3 for both the DIP and MCP (or minor and base, respectively) knuckles. Keeping these threshold values in view, the proportion of “suitable for identification” knuckle regions is calculated for the ground truth test subset.

Table 1 presents the values of these proportions for all three datasets for various object detectors along with the proposed ensemble approach of knuckle localization. It can be observed from the table that the proposed ensemble approach yields the highest proportion of knuckle regions which are “**suitable for identification**”, with more than 99% PIP and DIP (major and minor) suitable knuckle regions for the HD and 11k hand datasets, whereas the proportions of suitable PIP and DIP knuckles for the challenging HUQ dataset are 96.39% and 97.82%, respectively. These high values for the HUQ dataset highlight the effectiveness of the proposed knuckle localization approach with unconstrained hand dorsal images (i.e., images with less user cooperation). Furthermore, the proportions of suitable MCP (or base) knuckles for HD, 11k and HUQ datasets are 98.75%, 94.07% and 89.28%, respectively, which outperforms the other object detectors with reasonable margins. Nonetheless, the proportion of suitable knuckles for the MCP knuckles is relatively lower than their counterparts for PIP and DIP knuckles. This can be attributed to little prominence of creases in the MCP knuckles.

On the other hand, Table 2 furnishes the mean-IoU values for all individual detectors and the proposed ensemble approach. It is evident from the table that the proposed approach outperforms all other detector models and provides more relevant predictions with the highest **mean-IoUs** for all knuckle types and all three datasets. Higher values of mean IoU for a specific knuckle type would mean that the knuckles of that type are localized with increased assuredness.

### 4.2. Point-Form Analysis

**Figure 4:**
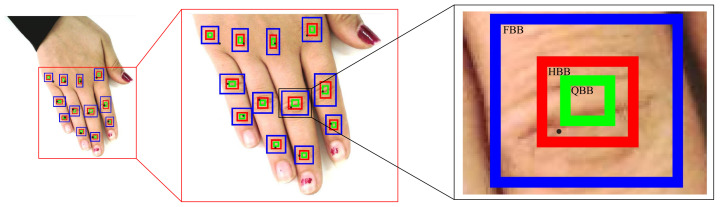
Illustration of calculating point-form metrics with black-dots as the detected keypoints from Openpose, blue-, red-, and green-colored boxes as the FBB, HBB, and QBB, respectively (refer to web version of the article for colored figures).

The point-form analysis is performed to evaluate the correctness of the centroids of the bounding boxes predicted from various object detectors. It is to ensure whether the centroid falls within the full bounding boxes (FBB), half bounding box (HBB), or quarter bounding box (QBB) of the ground truth boxes in increasing order of the strict sense of inclusiveness, respectively. The idea of FBB, HBB, and QBB is depicted more clearly in Figure 4. Any keypoint or centroid that falls within the respective box counts towards the calculation of FBB, HBB, or QBB accuracy. This analysis facilitates the comparison of the proposed approach with the state-of-the-art hand keypoint detector, called Openpose [17]. Moreover, it also offers to evaluate the appropriateness of the predicted boxes by checking their centroids, which cannot be otherwise checked through the box-form analysis.

For the numerical evaluation of the point form analysis, we define following important evaluation metrics, which would measure the preciseness of the detected keypoint to varying degrees.

1. *Full bounding box (FBB) accuracy*: This metric measures the accuracy of keypoints by just referring to their placements inside the full GTBB, i.e., any keypoint lying inside the GTBB is treated as correct.
2. *Half bounding box (HBB) accuracy*: This metric calculates the accuracy of the hand keypoints in terms of their placement inside the half-sized GTBB placed at the center, i.e., any keypoint lying inside the half-size GTBB is treated as correct.
3. *Quarter bounding box (QBB) accuracy*: This metric calculates the accuracy of the hand keypoints in terms of their placement inside the quarter-sized GTBB placed at the center, i.e., any keypoint lying inside the quarter-size GTBB is treated as correct.

In other words, the three evaluation metrics defined above provide the degree of preciseness of the detected keypoints in the increasing order. Figure 4 illustrates an instance where one of the detected keypoints (the PIP of middle finger) counts towards the FBB and HBB accuracy (as it falls within the respective boxes), but does not count towards the QBB accuracy (as it is outside the QBB box). Hence, it can be stated that the QBB accuracy is the most competitive among all metrics of the point-form analysis.

The comparison of point-form metrics for various object detectors, Openpose, and the proposed ensemble approach is presented in Table 3. It is not difficult to observe that the QBB accuracy has the least values for all types of detectors. Despite this, the proposed ensemble approach yields the best values for all three metrics and for all the datasets. The FBB accuracy values yielded by the proposed approach for the PIP (major) knuckles are 99.59%, 99.90%, and 98.53% for the 11k, HD, and HUQ datasets, respectively. These values are on par in comparison to their counterpart values for all other detectors. It is also apparent from the table that Openpose generates reasonable accuracy for knuckle localization with 11k and HUQ datasets (especially for the 11k dataset, where it is close to the second best value after the proposed approach). It is also worth specifying that the left hand images from 11k were horizontally flipped before executing the Openpose algorithm on them. This is to comply with the requirement of Openpose, where the input hand image is always considered to come from the right hand [17]. However, it was also observed that Openpose performs very poorly with the HD dataset which may be due to various reasons such as absence of discernible wrist, dark skin color, and brighter background. Furthermore, the proposed ensemble approach furnishes outperforming values for the QBB accuracy, which happens to be the toughest accuracy value. Notably, the state-of-the-art hand keypoint detector Openpose detects the knuckle keypoints with reasonable FBB accuracy. However, those keypoints seem to be off the quarter bounding boxes, as indicated by the consistent low values of QBB accuracy with Openpose for all three datasets. This is a rationale for the inference that the keypoints detected by the state-of-the-art Openpose tool are more likely to be away from the center of the ground truth boxes, which limits its applicability for knuckle localization in forensics applications.

**Figure 5:**
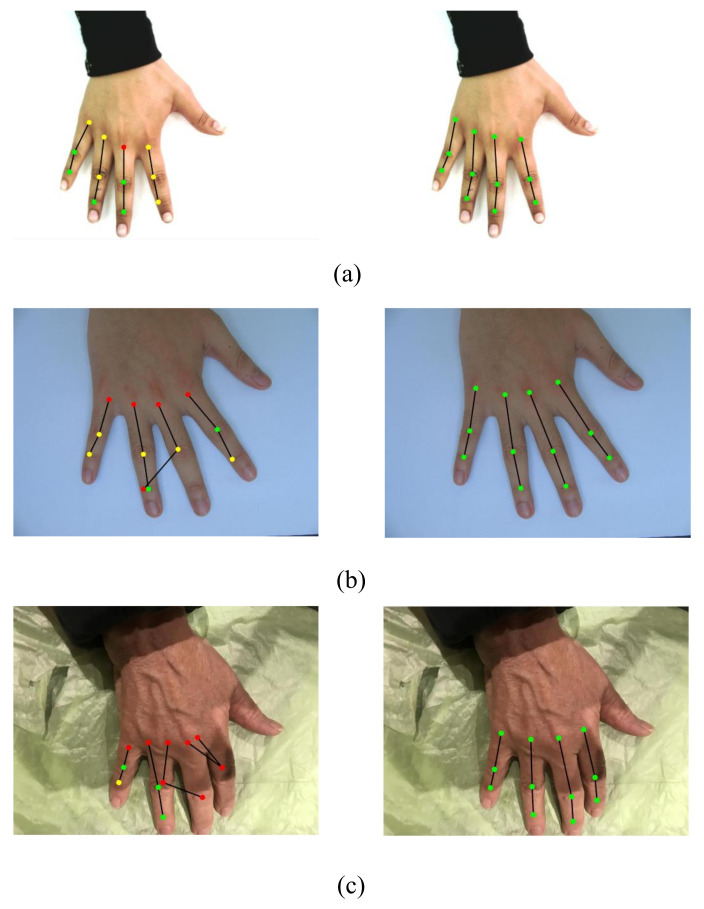
Samples showing finger skeletonization; Left column: Openpose; Right column: WBF; (**a**) 11k, (**b**) HD, and (**c**) HUQ dataset. Different colored markers correspond to different types of keypoints: Green: Falling within QBB; Yellow: Falling outside QBB but within FBB; and Red: Falling outside FBB (only knuckle keypoints are shown for Openpose) (refer to the web version for colors).

The comparison of skeletonization achieved by the state-of-the-art Openpose hand keypoint detector and the proposed WBF approach is presented in Figure 5. The figure demonstrates that Openpose works nicely with 11k and HUQ datasets, though the placement of keypoints detected by Openpose is not aligned with the knuckle crease region. This is the reason for the poor results of Openpose, especially the QBB accuracy. Additionally, performance of Openpose with the HD dataset is very poor. Openpose does not provide adequate skeletonization for the majority of the images from the HD dataset. The same is reflected from low values of point-form accuracy of Openpose with the HD dataset. On the other hand, the proposed knuckle localization framework performs strongly with all the three datasets, as is depicted in Figure 5. It also outperforms the Openpose algorithm for HD and the challenging HUQ datasets. Furthermore, the skeletonization achieved by the proposed approach indicates that the centroids of the predicted bounding boxes are placed nicely at or near to the centers of the knuckle crease region. This precisely supports the high values of point-form accuracy metrics of the proposed approach presented in Table 3.

### 4.3. Cross-Dataset Evaluation

In order to evaluate the generalizability of the proposed knuckle localization approach, we further perform the cross-dataset evaluation, were models trained on hand images from one database are tested with hand images from another database. This evaluation facilitates the generalizability study of the proposed approach and a more meaningful comparison against the state-of-the-art Openpose algorithm because both these testing environments constitute exclusive test data which are completely unknown for the concerned model. To make this comparison appropriate and fair, we test the models trained on the multi-ethnic HUQ database against the images of HD and 11k databases. For the better understanding of the readers, we adopt following nomenclature for the models for cross-dataset testing:

- Model M1: Trained on original right and left hand images from HUQ dataset.
- Model M2: Trained on original right and flipped left hand images from HUQ dataset (the flipping of left hand images is performed to ascertain the procedure fall in line with the procedure of state-of-the-art hand keypoint detector Openpose).

The results for cross-dataset evaluation of HD and 11k datasets using Model M1 are presented in Table 4 and Table 5, respectively. Results for the state-of-the-art Openpose hand keypoint detector are also included in these tables. It can be observed from Table 4 that the proposed ensemble based approach outperforms all individual detectors and Openpose in all possible cases. The poor performance of Openpose can be related with the imaging properties of the HD dataset, where wrist region is absent in the majority of the images, leading to misinterpretation of the hand keypoints. On the other hand, if we refer to Table 5, performance of the proposed approach is either superior or comparable to the individual detectors and Openpose. There is only one case (FBB accuracy for minor knuckles) where Openpose has outperformed the proposed approach significantly. This substantial performance of the proposed approach validates its generalizability to work across multiple datasets.

However, in order to align the cross-dataset experimentation more with the setup of Openpose, we consider another scenario of testing where all the left hand images are flipped in advance to appear as right hand images (named as Model M2). In this scenario, the training and testing tasks are performed with original right and flipped left hand images, similar to the Openpose algorithm [17]. This scenario of testing images reduces the complexity of the training task and allows comprehensive learning of the individual object detectors (though at the cost of one additional step of identifying left hand images and flipping them). The cross-dataset results for model M2 are presented in Table 6 and Table 7, respectively. It can be observed from these tables that model M2 aids in the overall results. Notably, it also improves the result for the proposed approach over Openpose in the specific case of FBB accuracy of minor knuckles from the 11k dataset, from 91.77% to 98.53%.

### 4.4. Matching Comparison

In order to establish the superiority of the proposed ensemble based knuckle localization approach, the matching experiment is performed with the knuckle regions extracted from all the detectors. All the knuckle localizations made in the manuscript are completed in the form of bounding box regression, and each bounding box is represented by the x- and y-coordinates of the top-left position and the associated width and height. Hence, all the predicted knuckle regions are extracted from the original hand image through rectangular crops, and these extracted knuckle regions are stored in order of the knuckle type and finger label they belong to. The transfer learning [30] is employed for the task of extracting distinguishing features. The pretrained DenseNet201 [31] model is employed for the transfer learning task, and to learn the fine-details of the extracted knuckle regions. The matching between feature vectors is performed through the Cosine distance and an “all-to-all” matching protocol. More details about the matching experiment can be found in [32]. Notably, the matching results are obtained by using the knuckle regions extracted from the WBF model trained on the HUQ dataset in “all-right hand” scenario. The mean equal error rates (mean-EERs) for various knuckle types of the two public databases are presented in Table 8.

The mean-EERs and mean-AUCs presented in Table 8 and Table 9 are calculated by taking average of the individual EERs and AUCs of all the fingers for each knuckle type. Table 8 demonstrates that the proposed ensemble based approach outperforms the individual detectors for each knuckle type for 11k datasets. Notably, the proposed approach yields a relative improvement of 16.20%, 5.07%, and 8.01%, respectively, for major, minor, and base knuckle types of 11k dataset with respect to the second best performing detector. On the other hand, mean-EERs for the HD dataset are also improved through the proposed ensemble approach of knuckle localization. In particular, mean-EERs for the major and base knuckle types of HD dataset improve by 19.16% and 8.73%, respectively, which is a significant improvement for real-world knuckle-matching scenario. However, the performance of WBF stands comparable to FRCNN101 for the minor knuckle types of HD dataset (with a marginal difference of 0.22%). The overall performance of the proposed approach shows a substantial improvement in the recognition capabilities of the system, which is validated through the mean-EER values for the whole hand for both the datasets. The best EER values considering the knuckle regions of the entire hand are reported to be 5.26% and 8.07% for HD and 11k datasets, respectively. The large values of percentage relative improvement turn out to be 7.88% and 18.41% for the HD and 11k datasets. The table once again highlights the need of the ensemble approach of localization, especially looking at the fact that the second best mean-EERs for HD and 11k datasets are not reported by any single object detector, hence accrediting the hypothesis of comprehensive localization through ensemble of multiple knuckle detectors algorithms.

On the other hand, Table 9 presents another important performance metric for biometrics matching, i.e., AUC. This table clearly demonstrates that the mean-AUC values for the proposed approach are the best among all detectors and for both the public datasets. The mean-AUC values for the HD dataset consistently fall in the range of 98.30–99.04%, which is a high AUC range, whereas the mean-AUC values for the 11k dataset is marginally lower than their counterparts for HD, especially with the base knuckles. Nevertheless, the proposed approach yields outperforming AUC values for 11k dataset. This is another evidence of how the matching performance can be improved by employing a more appropriate knuckle localization scheme, such as ours.

## 5. Discussion

**Figure 6:**
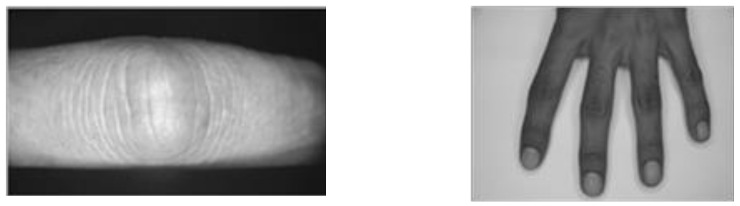
Sample of images taken from (**left**) [8], (**right**) [15].

It has been demonstrated that the performance of our approach can outperform the competing approaches for localizing the MCP, PIP, and DIP finger knuckle regions. Notably, there has been no work in the literature that report numerical evaluation of knuckle localization. However, certain studies established the automatic localization of knuckle regions [8,15,16]. The approaches in [8,15] were designed to handle the knuckle images from images of a single finger or half dorsal view (without clear base knuckles) of the hands, as depicted in Figure 6 (left) and (right), respectively. Therefore, these approaches were designed to work in a much more limited scenario without considering the full hand image or hand pose with touching fingers. Hence, they do not offer a direct comparison with the proposed approach.

On the other hand, the approach in [16] focused on localising the finger-valley keypoints for isolation of fingers and then used the finger geometry for knuckle localization. Therefore, these approaches cannot export comparable regions without further work. It also becomes cumbersome to detect the finger valley keypoints for the hand images having fingers closed. Moreover, all these known approaches which present automated knuckle localization do not perform any quantitative evaluation of the same, hence making it impossible to report any accuracy for the said problem of knuckle localization.

Additionally, the proposed approach can localize the knuckle centroid keypoints and knuckle regions for extraction and work with data having large variations. The results include evaluations from both the region localization (box-form) and center-point detection (point-form) framework. The proposed approach has excelled in both these frameworks hence establishing a single best approach to be employed with diverse datasets. This work has demonstrated that accurate localization of knuckle regions can lead to improvements in the performance of biometric matching experiments, which is illustrated through apposite performance metrics EER and AUC.

Beyond CNN, graph convolutional networks (GCNs) apply convolutions on graph structured data, which are usually more sparse, unlike unstructured data, and have been used to assist object detection [33,34]. GCN has been employed for applications such as action recognition, aerial images, and scene text recognition, but there is no reported work on knuckle localization. It may be worth considering the potential for GCN refinement for the current application. Transformers have also observed increasing utility in computer vision applications, including object detection [35,36]. However, they usually need costly pre-training on large datasets because they provide the convolutional inductive bias by performing self-attention across the patches of images, unlike CNN which leverages from the hard inductive bias in presence of low data. This requirement is a particular disadvantage for knuckle detection, because such large datasets do not yet exist.

## 6. Conclusions

This paper proposes an ensemble approach of knuckle localization, which leverages from multiple single-stage and two-stage state-of-the-art object detectors to perform classification and bounding box regression for various knuckle types available on the hand-dorsum. The ensemble approach is developed with the idea to have improved and more accurate localization of the knuckle regions, which can in turn make the whole recognition process inclusive and comprehensive without the need to refer a considerable portion of images. The proposed ensemble approach of knuckle localization builds over the capabilities of individual detectors and provide more apt bounding boxes for all three knuckle types (MCP, PIP, and DIP). The superior performance of the proposed approach is ascertained through exhaustive set of experiments on two large-scale public hand databases (11k and HD) and one realistic and challenging dataset (HUQ). The outperforming nature of the proposed approach is established through various suitable metrics which are evolved by keeping the two-fold requirement of the problem at hand. In view of this, the predicted knuckle regions are assessed through both the point- and box-form analysis to ensure the quality of overall bounding boxes and preciseness of their centroids, respectively. The paper is also furnished with the first-of-its-kind numerical evaluation of the knuckle localization, facilitating a justified comparison of various detectors and inviting more investigations in the said domain. Furthermore, the comparison of EER for various knuckle regions of the employed datasets reveal that the proposed approach generates outperforming results in the matching experiment too, leading to the development of an overall improved hand-biometric system. In the future, we will investigate the knuckle localization procedure for hand images in the 3D domain. Moreover, we would like to extend the investigation of the current approach to a greater number of unconstrained images having large variations in illumination, orientation, and background.

## Figures and Tables

**Table 1:** Proportion of “suitable for identification” knuckles.

Database	Knuckle	Yolov3	Yolov2	FR-CNN (resnet50)	FR-CNN (resnet101)	WBF
	**Major**	97.65%	81.03%	98.30%	98.50%	**99.55%**
**HD**	**Minor**	99.15%	86.67%	98.65%	99.65%	**99.95%**
	**Base**	95.35%	66.08%	91.17%	97.60%	**98.75%**
	**Major**	85.95%	43.91%	97.82%	92.75%	**99.08%**
**11k**	**Minor**	93.00%	59.69%	98.73%	97.92%	**99.39%**
	**Base**	88.49%	50.76%	91.53%	91.68%	**94.07%**
	**Major**	75.10%	61.69%	95.38%	92.58%	**96.39%**
**HUQ**	**Minor**	88.97%	81.40%	96.24%	96.29%	**97.82%**
	**Base**	75.00%	69.61%	84.20%	87.45%	**89.28%**

**Table 2:** Mean-IoUs.

Database	Knuckle	Yolov3	Yolov2	FR-CNN (resnet50)	FR-CNN (resnet101)	WBF
	**Major**	0.6417	0.6707	0.6959	0.7366	**0.7702**
**HD**	**Minor**	0.5461	0.5644	0.6944	0.6464	**0.7067**
	**Base**	0.3663	0.3464	0.4558	0.4369	**0.5054**
	**Major**	0.6255	0.4185	0.7233	0.6693	**0.7533**
**11k**	**Minor**	0.5119	0.3567	0.6325	0.6156	**0.6516**
	**Base**	0.5127	0.3151	0.5553	0.5687	**0.5975**
	**Major**	0.5723	0.5107	**0.7132**	0.6581	**0.7132**
**HUQ**	**Minor**	0.5055	0.4896	0.6525	0.6438	**0.6773**
	**Base**	0.4225	0.3991	0.4841	0.4924	**0.5167**

**Table 3:** Point-form comparison of different detectors.

Database	Detector	FBB-acc (%)			HBB-acc (%)			QBB-acc (%)		
		Major	Minor	Base	Major	Minor	Base	Major	Minor	Base
**11k**	**Yolo-v3**	98.17	96.19	91.47	88.22	67.76	64.18	44.95	20.59	25.36
	**Yolo-v2**	73.73	66.68	57.07	44.96	34.23	23.68	17.53	10.98	6.91
	**FRCNN50**	98.93	98.78	93.14	98.06	93.55	75.20	76.24	59.39	35.59
	**FRCNN101**	96.53	98.23	94.60	95.10	92.03	73.30	70.37	56.46	34.71
	**OpenPose**	98.83	97.51	56.20	81.16	60.85	14.54	32.72	21.65	2.89
	**WBF**	**99.59**	**99.39**	**95.37**	**99.08**	**95.22**	**78.17**	**85.98**	**62.71**	**41.22**
**HD**	**Yolo-v3**	99.65	99.50	96.75	97.70	88.60	75.50	66.50	43.10	33.15
	**Yolo-v2**	97.40	88.35	71.20	81.50	56.90	31.45	44.90	23.00	9.90
	**FRCNN50**	98.80	98.65	91.90	98.00	96.50	80.70	81.25	77.20	42.70
	**FRCNN101**	99.20	99.65	98.00	98.50	94.25	85.90	84.85	67.85	47.05
	**OpenPose**	23.61	23.71	9.82	20.09	13.54	1.44	9.92	3.62	0.25
	**WBF**	**99.90**	**100.00**	**99.10**	**99.40**	**97.80**	**90.30**	**92.20**	**78.90**	**52.85**
**HUQ**	**Yolo-v3**	94.00	92.33	80.79	76.27	64.13	40.85	32.57	20.63	12.45
	**Yolo-v2**	82.37	83.84	73.93	61.18	54.01	35.11	30.23	23.68	12.50
	**FRCNN50**	97.05	96.29	85.11	95.48	92.89	56.81	**79.01**	**65.55**	21.44
	**FRCNN101**	97.56	96.54	86.84	92.58	88.31	57.77	57.32	49.85	22.00
	**OpenPose**	93.19	88.41	67.58	67.53	50.00	28.51	26.17	17.68	7.98
	**WBF**	**98.53**	**97.92**	**89.48**	**95.88**	**93.14**	**59.76**	71.34	60.52	**23.32**

**Table 4:** Cross-dataset evaluation of HD dataset with model M1.

Detector	FBB-acc (%)			HBB-acc (%)			QBB-acc (%)		
	Major	Minor	Base	Major	Minor	Base	Major	Minor	Base
**Yolo-v3**	92.86	88.44	75.74	79.46	55.16	48.91	36.41	19.94	18.20
**Yolo-v2**	87.00	66.17	52.38	67.81	35.22	15.28	32.54	13.29	3.87
**FRCNN res50**	95.54	90.48	78.52	94.99	83.93	62.35	86.31	56.10	28.82
**FRCNN res101**	99.50	98.51	81.60	97.97	93.30	56.80	76.29	63.14	21.97
**OpenPose**	23.61	23.71	9.82	20.09	13.54	1.44	9.92	3.62	0.25
**WBF**	**99.50**	**98.71**	**92.26**	**98.96**	**95.14**	**70.98**	**86.71**	**68.60**	**32.34**

**Table 5:** Cross-dataset evaluation of 11k dataset with model M1.

Detector	FBB-acc (%)			HBB-acc (%)			QBB-acc (%)		
	Major	Minor	Base	Major	Minor	Base	Major	Minor	Base
**Yolo-v3**	94.46	93.70	88.11	74.64	56.20	51.47	32.93	18.70	19.00
**Yolo-v2**	66.11	56.61	42.58	37.35	25.76	16.26	13.62	7.11	4.07
**FRCNN res50**	97.92	89.68	88.92	**97.36**	86.53	62.91	**80.84**	57.57	28.20
**FRCNN res101**	95.93	89.08	92.33	92.07	82.01	63.97	71.24	53.66	28.40
**OpenPose**	98.83	**97.51**	56.20	81.16	60.85	14.54	32.72	21.65	2.89
**WBF**	**99.09**	91.77	**93.95**	96.90	**87.55**	**67.43**	79.93	**59.91**	**32.32**

**Table 6:** Cross-dataset evaluation of HD dataset with model M2.

Detector	FBB-acc (%)			HBB-acc (%)			QBB-acc (%)		
	Major	Minor	Base	Major	Minor	Base	Major	Minor	Base
**Yolo-v3**	94.05	90.43	72.27	75.74	56.50	44.39	29.51	20.34	16.22
**Yolo-v2**	91.96	69.59	53.27	74.01	37.15	14.78	41.07	12.80	3.27
**FRCNN res50**	97.77	96.33	71.08	97.22	88.84	52.78	86.01	50.10	24.01
**FRCNN res101**	99.26	99.21	93.11	98.71	91.32	67.76	84.47	60.91	27.98
**OpenPose**	23.61	23.71	9.82	20.09	13.54	1.44	9.92	3.62	0.25
**WBF**	**99.90**	**99.21**	**94.54**	**99.40**	**92.96**	**70.73**	**90.92**	**65.38**	**30.80**

**Table 7:** Cross-dataset evaluation of 11k dataset with model M2.

Detector	FBB-acc (%)			HBB-acc (%)			QBB-acc (%)		
	Major	Minor	Base	Major	Minor	Base	Major	Minor	Base
**Yolo-v3**	93.90	94.21	87.80	72.51	60.82	49.95	30.69	23.83	18.55
**Yolo-v2**	65.60	59.15	49.14	35.26	27.39	18.80	13.31	8.03	4.67
**FRCNN res50**	98.32	96.75	87.60	97.41	92.17	62.40	70.88	53.81	26.17
**FRCNN res101**	97.36	96.85	93.29	95.99	89.79	65.09	72.76	54.32	26.98
**OpenPose**	98.83	97.51	56.20	81.16	60.85	14.54	32.72	21.65	2.89
**WBF**	**99.24**	**98.53**	**94.61**	**98.22**	**93.45**	**70.27**	**82.42**	**60.82**	**30.54**

**Table 8:** Comparison of mean-EER for the public databases.

Databases→	HD				11k			
**Detector↓**	Major	Minor	Base	Hand	Major	Minor	Base	Hand
**yolov3**	6.88%	7.89%	14.69%	9.82%	7.96%	9.64%	11.61%	9.74%
**yolov2**	6.97%	8.60%	8.68%	8.08%	14.12%	16.46%	14.84%	15.14%
**FRCNN50**	6.22%	6.47%	16.80%	9.83%	6.11%	8.86%	18.16%	11.04%
**FRCNN101**	5.01%	**5.24%**	6.87%	5.71%	7.48%	9.36%	13.92%	10.25%
**WBF**	**4.05%**	5.46%	**6.27%**	**5.26%**	**5.12%**	**8.41%**	**10.68%**	**8.07%**

**Table 9:** Comparison of mean-AUC for the public databases.

Databases→	HD				11k			
**Detector↓**	Major	Minor	Base	Hand	Major	Minor	Base	Hand
**yolov3**	97.75%	96.66%	90.20%	94.87%	96.84%	96.29%	94.55%	95.89%
**yolov2**	97.92%	96.79%	96.84%	97.18%	93.23%	91.81%	93.20%	92.75%
**FRCNN50**	97.88%	97.94%	87.90%	94.57%	98.07%	96.83%	87.00%	93.97%
**FRCNN101**	98.17%	98.48%	97.24%	97.96%	96.59%	96.78%	92.18%	95.18%
**WBF**	**99.04%**	**98.87%**	**98.30%**	**98.74%**	**98.93%**	**97.36%**	**95.66%**	**97.32%**

## Data Availability

The public hand databases 11k and HD are respectively available at: https://sites.google.com/view/11khands (accessed on 21 December 2021) and http://www4.comp.polyu.edu.hk/~csajaykr/knuckleV2.htm (accessed on 21 December 2021). The proprietary HUQ database (which is specifically collected under the H-Unique project) is still under collection.
